# Congenital Absence of the Left Pulmonary Artery Associated With Right‐Sided Aortic Arch Presenting as Hemoptysis in an Adult: A Case Report

**DOI:** 10.1002/ccr3.73431

**Published:** 2026-09-16

**Authors:** Tayyaba Kauser, Asma Khan, Asma Sattar, Syed Muhammed Salman Hassan, Masroor Raza, Sarim Hassan Shahab, Fazal Habib

**Affiliations:** ^1^ Tree Top Hospital Dhum'buri Magu Maldives; ^2^ Nishtar Medical University Multan Pakistan; ^3^ Sher‐e‐Bangla Medical College Barisal Bangladesh

**Keywords:** congenital vascular anomaly, hemoptysis, pulmonary artery absence, right‐sided aortic arch, unilateral pulmonary artery agenesis

## Abstract

Congenital unilateral absence of the pulmonary artery is a rare vascular anomaly that may remain undiagnosed until adulthood. Patients can present with unexplained hemoptysis, recurrent respiratory infections, or pulmonary hypertension. Contrast‐enhanced CT plays a crucial role in diagnosis, evaluation of collateral circulation, and guiding appropriate therapeutic management.

## 1 Introduction

Congenital Absence of a Pulmonary artery is a rare vascular anomaly resulting from abnormal development of the sixth aortic arch, with failure of the proximal sixth aortic arch to connect normally with the pulmonary trunk [1]. Historically, 67% of cases are due to agenesis in the right pulmonary artery, although the distribution varies among patient populations and according to the presence of associated congenital heart disease. Absence of the left pulmonary artery is less frequently reported. It causes hypoperfusion and hypoplasia of the affected lung. The estimated prevalence is 1 in 200,000. It can also be associated with cardiovascular anomalies. These include variations in the anatomy of the aortic arch, such as a right‐sided aortic arch with variant branching patterns [2].

Patients may remain asymptomatic or present with hemoptysis, dyspnea during exertion, chest pain or recurrent pulmonary infections. Affected lung is supplied by systemic collateral vessels which can lead to pulmonary hypertension and life‐threatening hemoptysis [3]. Cross‐sectional imaging, particularly contrast‐enhanced computed tomography, can effectively visualize vascular anatomy and collateral circulation.

We report a case of a 55‐year‐old male who presented with hemoptysis and was diagnosed with congenital absence of left pulmonary artery with right‐sided aortic arch.

## 2 Case History

Our patient, a 55‐year‐old male, presented with a complaint of hemoptysis. He reported expectorating blood‐stained sputum associated with mild cough, which had begun approximately 1 week prior to presentation. The hemoptysis was intermittent and not associated with large‐volume blood loss.

Upon retrospective questioning, the patient recalled having experienced two episodes of respiratory tract infections over the past year. These episodes were mild and were treated symptomatically without formal diagnostic workup. He also admitted to occasional mild chest pain, predominantly on exertion. However, the chest pain was not severe and did not significantly limit his activities.

Notably, the patient denied any significant prior medical history and his general health had been good until the recent onset of hemoptysis. He had no prior history of tuberculosis, asthma, chronic bronchitis, or heart disease.

On physical examination, his vital signs were within normal limits. Chest auscultation revealed wheeze localized to the left lung. His complete blood count was within normal limits. Post‐contrast CT chest was performed.

## 3 Investigations

**FIGURE 1:**
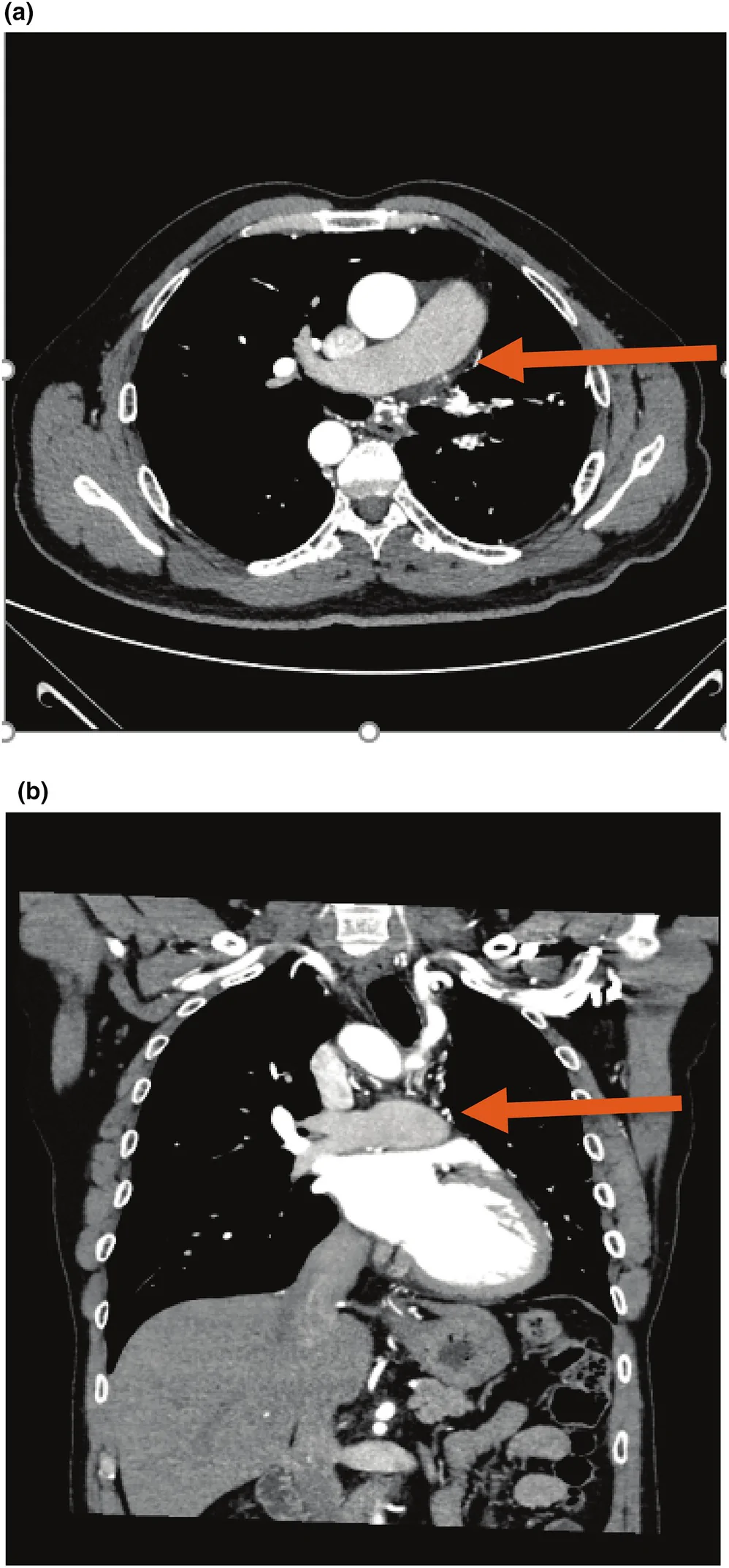
(a) and (b) Axial and coronal images showing absent left pulmonary artery.

**FIGURE 2:**
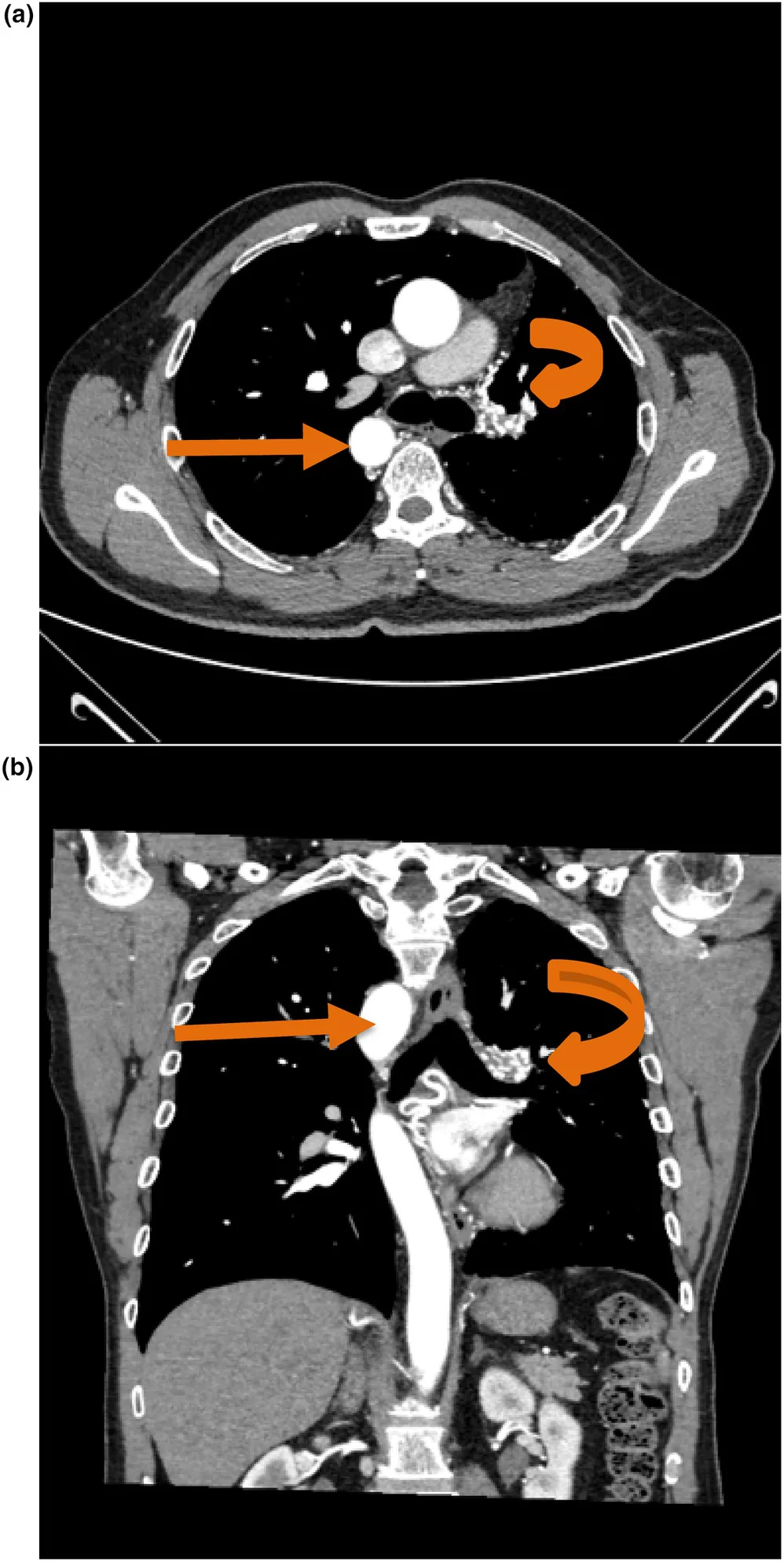
(a) and (b) Axial and coronal images showing right‐sided aortic arch (straight arrows) and collateral formation to supply left lung (curved arrows).

**FIGURE 3:**
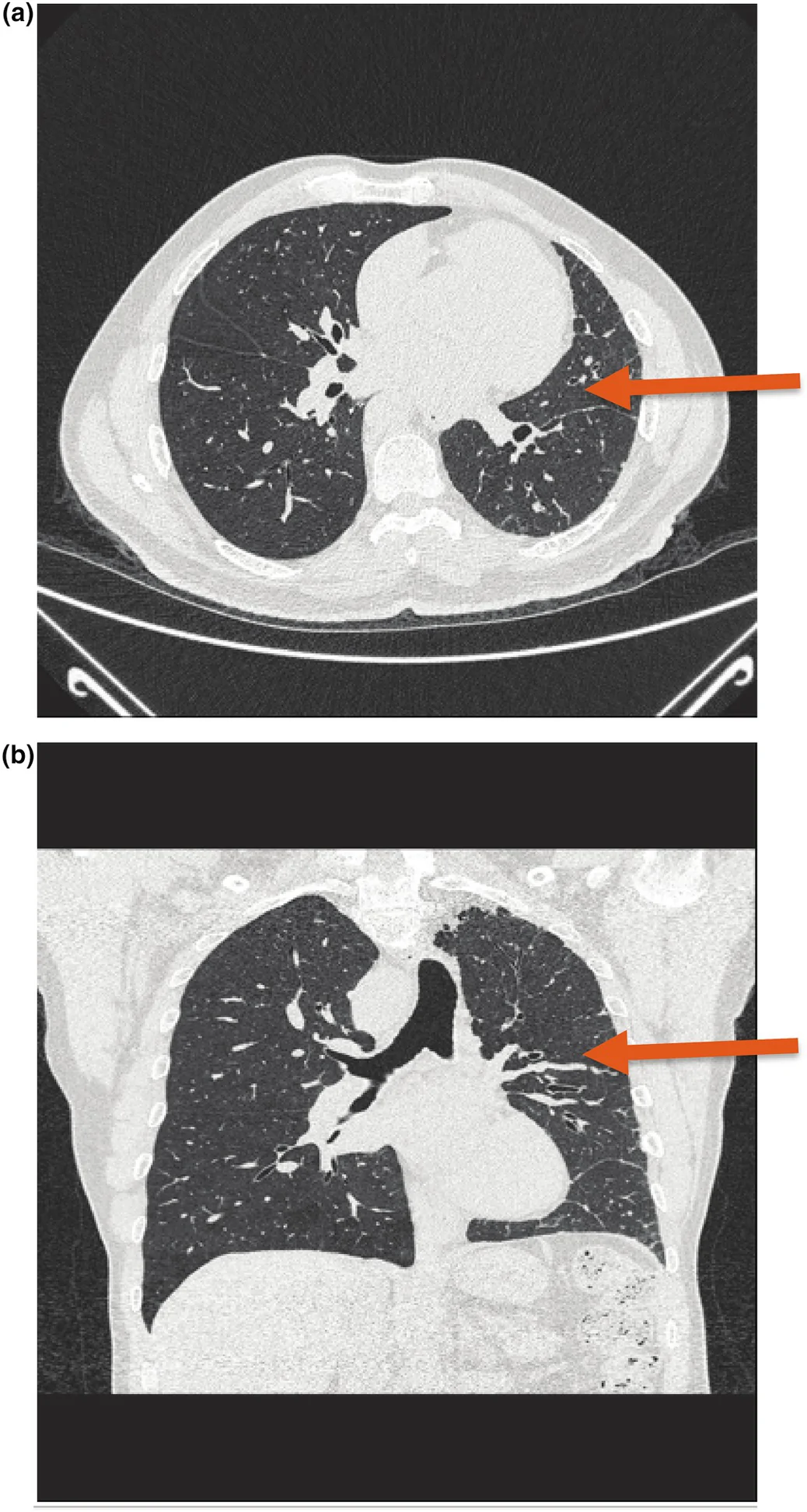
(a) and (b) Axial and coronal images lung window images showing mild left lung volume loss.

Our patient's chest CT demonstrated absence of the left pulmonary artery (Figure 1), with a reduced‐volume left lung supplied by multiple systemic collateral vessels, associated with compensatory hyperinflation of the right lung and mild mediastinal herniation. The left lung also showed smooth interlobular septal thickening and emphysematous changes, likely reflecting post‐inflammatory or chronic hypoperfusion‐related changes. A right‐sided aortic arch with a variant branching pattern was noted (Figures 2 and 3).

Transthoracic echocardiography was performed which did not reveal any additional cardiac anomalies. There were no echocardiographic signs of pulmonary hypertension.

## 4 Management and Follow‐Up

Given the absence of massive or life‐threatening hemoptysis, hemodynamic stability, and lack of echocardiographic evidence of pulmonary hypertension, the patient was managed conservatively. He was advised regular clinical follow‐up with periodic echocardiography to monitor for the development of pulmonary hypertension. At last follow‐up, he remained clinically stable with no recurrence of hemoptysis.

## 5 Differential Diagnosis

The differential diagnosis for unilateral absent or hypoplastic pulmonary artery with a small ipsilateral lung includes hypogenetic lung syndrome (scimitar syndrome), Swyer–James–MacLeod syndrome, and chronic pulmonary embolism. Hypogenetic lung syndrome is characterized by right lung hypoplasia associated with anomalous pulmonary vein draining the right lung into systemic circulation, features that were not present in this case. Swyer–James–MacLeod syndrome represents a post‐infectious obliterative bronchiolitis and is usually associated with a hyperlucent lung and air trapping rather than complete absence of the pulmonary artery. Chronic pulmonary embolism may result in a markedly reduced or non‐opacified pulmonary artery; however, in contrast to congenital absence, the proximal pulmonary artery is present, often appearing as a narrowed vessel contiguous with the main pulmonary artery. Intraluminal thrombus, eccentric wall thickening, webs, bands, or calcifications may also be seen in the affected pulmonary artery.

## 6 Discussion

Unilateral pulmonary artery agenesis (UPAA) is a rare congenital vascular anomaly. It was first diagnosed in 1868 by Fräntzel, a German physician [4]. It has a prevalence of 1 in 200,000 individuals. It occurs between the fifth and sixth weeks of gestation due to malformation in the sixth aortic arch [5]. Failure of normal development of the sixth aortic arch and its connection with the pulmonary trunk results in interruption or absence of the proximal pulmonary artery. As a result, the proximal artery is absent, while the distal intrapulmonary arterial branches may persist as hypoplastic vessels which are supplied by systemic collateral circulation.

UPAA can occur unilaterally or rarely bilaterally. In 67% of cases, the right pulmonary artery is absent, whereas left‐sided agenesis is less common and more frequently associated with additional cardiovascular anomalies such as a right‐sided aortic arch. Other associated malformations include tetralogy of Fallot, ventricular septal defect, and transposition of great vessels. There are approximately 420 cases documented in literature [6]. Most cases can be diagnosed and surgically treated in the first year of life if cardiovascular anomalies are present [7]. However, asymptomatic infants may not be diagnosed until adulthood, when the abnormality might be incidentally discovered on imaging or the patient may present with hemoptysis, recurrent respiratory infections, dyspnea, or pulmonary hypertension.

The clinical presentation is highly variable. Approximately 40% of patients present with exertional dyspnea, 20% develop hemoptysis or pulmonary hypertension. Other presentations include chest pain, pleural effusion, and recurrent pulmonary infections [8]. Our patient's presentation with hemoptysis in the absence of any cardiopulmonary disease highlights the subtle and overlooked nature of this disease in adults.

In the absence of a normal pulmonary arterial supply, the affected lung receives blood from bronchial (70%), phrenic artery (50%), intercostal (40%), and internal thoracic arteries (40%). These collateral vessels eventually hypertrophy and rupture, resulting in hemoptysis. Chronic hypoperfusion of the affected lung also results in pulmonary hypoplasia, reduced vascularity, and impaired mucociliary clearance, which predisposes patients to recurrent infections. Additionally, increased blood flow through the contralateral pulmonary artery may result in pulmonary hypertension.

It is challenging to diagnose UPAA due to nonspecific clinical symptoms. In patients who present with unexplained hemoptysis or recurrent respiratory infections and a high index of suspicion is present, a complete medical history and assessment should be done. Multiple imaging modalities are used in conjunction. Chest radiography is usually performed as the first diagnostic step and may reveal findings such as a small ipsilateral hemithorax, mediastinal shift toward the affected side, absence of hilar vasculature, diminished pulmonary vascular markings, and compensatory hyperinflation of the contralateral lung.

Definitive diagnosis is established using cross‐sectional imaging techniques such as contrast‐enhanced computed tomography (CT) or magnetic resonance imaging (MRI). These modalities allow direct visualization of the absent pulmonary artery. Additional findings include mosaic attenuation patterns, reduced pulmonary vasculature, and hypertrophied systemic collateral vessels. Our patient chest CT revealed absence of the left pulmonary artery, with a small volume left lung being perfused by multiple systemic collaterals, right‐sided aortic arch with variant branching pattern, and emphysematous changes in left lung. Compensatory hyperinflation of the right lung with mild mediastinal herniation was also present. Transthoracic echocardiography is used to assess pulmonary artery pressures and detect associated cardiac anomalies. Although pulmonary angiography remains the gold standard for diagnosis, it is now rarely required due to advances in noninvasive imaging.

### 6.1 Comparison With Previously Reported Adult Cases

Comparison with previously reported adult cases highlights the variability in presentation and management of UPAA. A 67‐year‐old man with left‐sided pulmonary artery agenesis presented with massive hemoptysis; detailed angiography was used to plan tailored treatment, and observation was ultimately chosen, with transcatheter arterial embolization (TAE) or surgery reserved for recurrence [9]. In contrast, a 35‐year‐old man with left‐sided agenesis complicated by hemoptysis required pneumonectomy, and an adult female with right‐sided agenesis who developed massive haemoptysis 4 years after diagnosis also underwent pneumonectomy, complicated by a bronchopleural fistula [10]. Milder, self‐limiting presentations have also been described: a 24‐year‐old man with left‐sided UPAA and a repaired ventricular septal defect presented with hemoptysis and cough [11], while a 55‐year‐old woman with right‐sided UPAA presented instead with exertional dyspnoea and pulmonary hypertension, managed conservatively with oxygen and pulmonary vasodilator therapy [12]. Our patient presented with mild hemoptysis without pulmonary hypertension, consistent with indolent end of the disease spectrum. This presentation supports conservative management with close follow‐up as a reasonable initial strategy in hemodynamically stable adults without evidence of pulmonary hypertension, reserving TAE or surgery for recurrent or massive bleeding.

There are no guidelines on the assessment or management of congenital absence of a pulmonary artery in adults due to its rarity. Therapeutic plans are individualized based on the patient's severity of presentation. Asymptomatic patients without cardiopulmonary dysfunction may be managed conservatively with regular follow up and annual echocardiography.

In symptomatic patients, particularly those with massive hemoptysis and recurrent respiratory tract infections or pulmonary hypertension, treatment is necessary. Transcatheter arterial embolization (TAE) is a preferred approach for controlling hemoptysis [13]. This technique involves selective occlusion of hypertrophied systemic collateral vessels and has favorable outcomes. It is performed under fluoroscopic guidance using a percutaneous femoral arterial approach via the Seldinger technique. A French catheter is used to identify collaterals, and a microcatheter is used to super selectively embolize the feeding vessels and minimize embolization of non target vessels. Embolic agents such as polyvinyl alcohol particles, microspheres, coils, or gelatin sponge are used. For distal occlusion, particles are used, whereas coils are preferred for proximal vessel control.

Although TAE is highly effective, recurrence may occur due to recruitment of new collateral vessels. Surgical intervention may be necessary in patients with recurrent hemoptysis.

Surgical options include pneumonectomy, lobectomy, and revascularization procedures. Pneumonectomy involves removal of the affected lung and may treat complications such as recurrent infections, hemoptysis, and pulmonary hypertension [14]. Based on our review of the prior literature, there have only been a handful of reports to date of surgical intervention involving pneumonectomy. Revascularization procedures are more successful in pediatric populations and are generally not feasible in adults [15]. It involves utilizing autologous pericardium or artificial materials for vascular reconstruction. Systemic‐pulmonary shunts are created by a modified Blalock‐Taussig shunt or catheterization [16], which promotes the growth of pulmonary vessels and the development of the lung. Subsequent corrective surgery is also done to reconstruct the absent PA.

Long term vasodilator therapy such as calcium channel blockers, phosphodiesterase 5 inhibitors, or prostacyclin analogs may be used to improve pulmonary hypertension and enhance survival [17]. In refractory cases, combined heart lung transplantation may be considered, although this is a last resort option.

The prognosis of UPAA depends on the severity of complications. While many patients remain stable for years, the development of hemorrhage or pulmonary hypertension can have adverse outcomes. The reported mortality rate is approximately 7% [18].

This case emphasizes the need of considering congenital vascular anomalies such as unilateral absence of the pulmonary artery in the differential diagnosis of hemoptysis even in patients with no prior significant medical history. Early recognition by advanced imaging is crucial for timely management and regular assessment should be done periodically to prevent complications. Patient education is also important to inform them about their condition, symptoms to watch out for, and the importance of follow up care.

## 7 Conclusion

In conclusion, Congenital unilateral absence of the pulmonary artery is a rare condition. It is usually asymptomatic; however, it can be symptomatic in childhood when associated cardiovascular anomalies are present which require prompt childhood intervention. Adult cases remain undiagnosed until later in life when they present with unexplained hemoptysis, lung infections, or pulmonary hypertension. This disease should be kept in the differential diagnoses of patients presenting with these symptoms even without a past medical history. Early diagnosis by advanced imaging and appropriate treatment is crucial to avoid serious complications.

## Author Contributions

**Tayyaba Kauser:** conceptualization, investigation, writing – original draft. **Asma Khan:** writing – original draft, investigation, data curation. **Syed Muhammed Salman Hassan:** writing – review and editing, investigation. **Asma Sattar:** writing – original draft, investigation, visualization. **Masroor Raza:** writing – review and editing, investigation. **Sarim Hassan Shahab:** writing – review and editing. **Fazal Habib:** writing – review and editing.

## Funding

The authors have nothing to report.

## Ethics Statement

The authors have nothing to report.

## Consent

Written informed consent was obtained from the patient for publication of this case report and accompanying images.

## Conflicts of Interest

The authors declare no conflicts of interest.

## Data Availability

The authors have nothing to report.
